# Editorial: Efficient deep neural network for intelligent robot system: Focusing on visual signal processing

**DOI:** 10.3389/fnbot.2023.1191655

**Published:** 2023-04-13

**Authors:** Xiao Bai, Xin Ning, Praveen Kumar Donta, Weijun Li

**Affiliations:** ^1^School of Computer Science and Engineering, State Key Laboratory of Software Development Environment, Jiangxi Research Institute, Beihang University, Beijing, China; ^2^Institute of Semiconductors, Chinese Academy of Sciences, Beijing, China; ^3^Distributed Systems Group, TU Wien (Vienna University of Technology), Vienna, Austria

**Keywords:** deep neural network, intelligent robot system, image processing, pattern recognition, visual navigation

With the availability of a vast amount of data in the public domain and the advancement of computing power, deep neural network (DNN) models are being increasingly utilized in machine learning tasks related to visual signal processing. However, the extensive use of larger DNN model architectures to enhance learning abilities and performance indicators for various tasks is hindered by their high complexity and computing power requirements. This impedes their efficient deployment on edge platforms and for real-time operations. To overcome these challenges and enable widespread deployment of DNNs in intelligent robot systems, researchers are now focusing on developing efficient DNN models that can improve their training and running speeds.

The purpose of this Special Issue is to collect high-quality articles on the recent development and trend of efficient DNN for intelligent robot system based on visual signal processing, and disseminate the outcomes and products from this topic to a wide range of communities, helping peers and non-expert readers understand the highly efficient design of DNNs. Researchers from all over the world actively participate and contributed a lot of manuscripts. After carefully and professionally reviewing all submissions, 12 high-quality manuscripts are accepted.

One contribution in this topic is about model pruning. Wu et al. propose a novel filter pruning method based on filter similarity to address the limitations of current criterion-based methods used for inference acceleration and hardware compatibility. It achieves significant FLOPs and parameter reduction with no loss in accuracy on different benchmark datasets and network architectures.

Six contributions are about the research of lightweight models and algorithms for classical image processing and computer vision tasks. Lan et al. propose a physical-model guided self-distillation network (PMGSDN) for single image dehazing. Experimental results on synthetic and real-world images show that the proposed method outperforms other methods and achieves high-quality dehazed results with clear textures and good color fidelity. Kumari and Mustafi develop a robust digital watermarking algorithm that uses an informed watermark retrieval architecture, fractional Fourier transform, blind source separation, and a heuristic algorithm. The algorithm's performance is evaluated against common attacks such as JPEG compression and Gaussian noise, and the optimal fractional domain is found using a genetic algorithm. Dai et al. propose SiamHFFT, a lightweight object tracking algorithm capable of handling small targets in complex scenarios. The proposed algorithm uses a hierarchical feature fusion transformer to extract multi-level features from a lightweight backbone, which allows for comprehensive feature representations in an end-to-end manner. The model achieves state-of-the-art results on various benchmark datasets and operates at a rate of 29 FPS on a CPU, making it practical for real-world applications. Zhong et al. propose a lightweight facial expression recognition model based on the Northern Goshawk Optimization algorithm and the bidirectional LSTM neural network, which improves recognition accuracy and can be effectively applied to facial expression recognition. Lu et al. present a facial image inpainting method using a multistage GAN and the global attention mechanism. The proposed method can effectively restore incomplete facial images by enhancing feature mining and semantic expression, using skip connections, encoder-decoder structure, and a local refinement network. Comparative experiments demonstrate that the proposed method generates realistic inpainting results with high PSNR and SSIM, indicating the model's performance and efficiency. Lin et al. address the shortage of boxing coaches in Chinese campuses by proposing a novel solution that employs human pose estimation technology to train interns. Specifically, they develop a model transfer technique that utilizes channel patching to enhance the accuracy of pose key points by an average of 1–20% and 3D accuracies by 0.3–0.5% compared to 2D baselines. The proposed method is not only practical but also effective for boxing pose estimation.

Five contributions focus on the implementation of lightweight models to address other signals. Zheng presents a writing feature abstraction process based on ON-LSTM and attention mechanism for sentiment analysis, addressing the problem of ignoring syntactic and tag semantics information in emotional text feature extraction. The study shows the high application potential of deep learning models for dynamic user sentiment analysis. Wang and Chen investigate teachers' acceptance of robotics education and its relationship to the effectiveness and sustainability of robotics education using the UTAUT model and deep learning algorithms. The study also found that deep learning models such as mDAE and AmDAE reduced training time compared to existing noise-reducing autoencoder models. Teng et al. address the lack of technical and algorithmic support in music therapy for cancer patients and design a neural network robotic system based on breast cancer patients to analyze the effect of music relaxation training on alleviating adverse reactions after chemotherapy. The research provides reference for the next development of neural network robot system in the medical field. Chen and Fan utilize the neural Turing machine model to investigate the tensile properties of metallic materials, and they improve the model to achieve faster and more explicit results. The improved model demonstrates potential for practical applications in the exploration of metal material tensile properties testing technology. Xue et al. develop a modular system for robots to collaborate with humans in using tools. The system uses a multi-layer instance segmentation network to find task-related operating areas and identify tools based on the state of the robot in the collaborative task, generating a state semantic region. The system performs well on an untrained real-world tool dataset and is validated using a robot platform based on Sawyer.

Overall, all papers published in this Special Issue show that efficient DNN for intelligent robot system have developed very fast in recent years. We hope that this topic can provide some references and novel ideas for researchers in this field. It should be emphasized that for such a rapidly developing research field, the work that has been done so far is only a drop in the ocean. The manuscripts we collect this time can only be a small leaf in the Amazon rainforest.

We would like to thank all the authors for their innovative contributions, and all the reviewers for their professional, crucial, yet constructive comments. Also, we wish to express our thanks to Mr Hang Ran, PhD students at Institute of Semiconductors, Chinese Academy of Sciences, for his assistance in this process. Last, we wish to express our gratitude to the editorial team of *Frontiers in Neurorobotics* for their support throughout this venture. We hope you enjoy this collection of papers and that the Special Issue can stimulate further research and development in this area.

## Author contributions

All authors listed have made a substantial, direct, and intellectual contribution to the work and approved it for publication.

